# Abdominal Wall Abscess Secondary to Cholecystocutaneous Fistula via Percutaneous Cholecystostomy Tract

**DOI:** 10.7759/cureus.4444

**Published:** 2019-04-12

**Authors:** Daniel H Lofgren, Sugam Vasani, Victorico Singzon

**Affiliations:** 1 Otolaryngology, McLaren Oakland Hospital, Pontiac, USA; 2 General Surgery, United Hospital Center, Bridgeport, USA; 3 Family Medicine, United Hosptial Center, Bridgeport, USA

**Keywords:** cholecystocutaneous fistula, percutaneous cholecystostomy, abscess, cholecystectomy, abdominal pain, non-compliance

## Abstract

Cholecystocutaneous fistulas (CCFs) are an increasingly rare consequence of chronic gallbladder inflammation and disease. Historically, they were commonly noted in the literature by Courvoisier, Naunyn, and Bonnet in the late 1800s. Due to improvements in diagnostic imaging and treatment options in the last century, there has been a marked decrease in the incidence of the CCF cases in the literature. From the late 1890s to 1949, there were only 37 cases presented in the literature; only 28 cases have been reported since 2007. This case is only the second noted CCF in the literature that followed percutaneous cholecystostomy drain placement and removal.

General surgery was consulted on a 60-year-old morbidly obese female, who presented to the emergency department after one week of fever, right upper quadrant (RUQ) pain, nausea, emesis, and shortness of breath. She had a history of acute cholecystitis treated with a cholecystostomy tube the year prior, but after the removal of the tube, she was lost to follow up. She was found to have a 14cm x 5cm fluctuant abdominal wall abscess in her RUQ that was treated with incision and drainage (I&D) along with ertapenem. She continued to improve until day 7 post-I&D when yellowish-green discharge was noted draining from the wound. After a negative hepatobiliary iminodiacetic acid scan, a follow-up abdominal computed tomography (CT) showed a contracted gallbladder with fistula formation underlying the abscess location, near the site of her prior cholecystostomy tube. A robotic-assisted cholecystectomy was performed, which improved the wound drainage, and the patient was discharged home 5 days later.

This case is the only noted CCF presenting as a RUQ abscess after cholecystostomy drain placement. The patient lacks follow up after the removal of her percutaneous drain and continued inflammation in the gallbladder provided perfect nidus for the fistula formation. As seen in other CCF patients, cholecystectomy is the treatment of choice, and this case was successfully treated via robotic-assisted cholecystectomy with adhesiolysis.

## Introduction

Biliary fistulas are pathologic connections formed from chronic inflammation between the gallbladder and adjacent organs [1-2]. Historically, biliary fistulas were a common outcome of longstanding gallbladder inflammation and disease [3-5]. With improving diagnostic technology and treatment, biliary fistulas are being reported to a lesser extent in the literature [6]. External biliary fistulas (EBFs), which generally connect the gallbladder to the skin, subcutaneous tissues, and abdominal wall, are increasingly rare in occurrences. A cholecystocutaneous fistula (CCF) connects the gallbladder to the skin, and is a form of EBF that has been reported with less frequency in the last century due to improving medical diagnostic techniques and identification. A total of 169 cases were reported by Courvoisier in the 19th century [7-8]. From 1900 to 1991, there were only a reported 65 cases of EBF in the literature [4,9-10]. From 1890 to 1949, only 37 cases were found in the medical literature. Since 2007, only 28 cases have been published [5-6]. These fistulas can be secondary to abdominal procedures or other traumatic causes. The cutaneous openings can drain bile and gallstones through external sinuses and have been noted at various locations including the right upper quadrant, umbilicus, and even in the gluteal folds [7,11]. Even more uncommon are the EBFs associated with percutaneous cholecystostomy drains, with only one other case report seen currently in the literature [12]. The case below is the first noted CCF presenting as an abscess following percutaneous cholecystostomy drain removal.

## Case presentation

**Figure 1:**
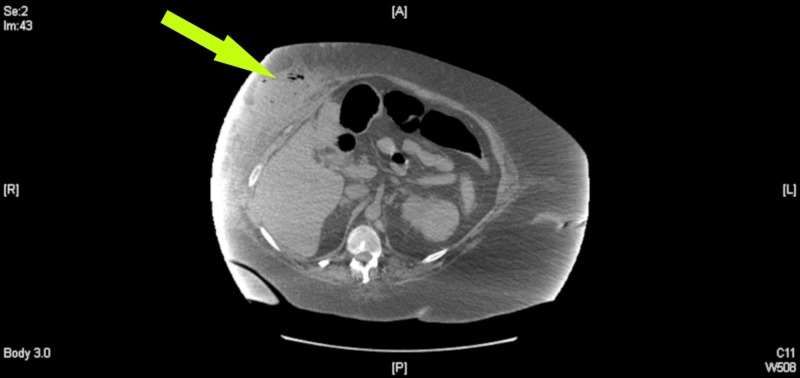
Axial view of noncontrast computed tomography of the abdomen. Arrow demonstrates subcutaneous right upper quadrant fluid collection with associated air-fluid levels and local fat stranding. Superiormost image of the abscess site.

**Figure 2:**
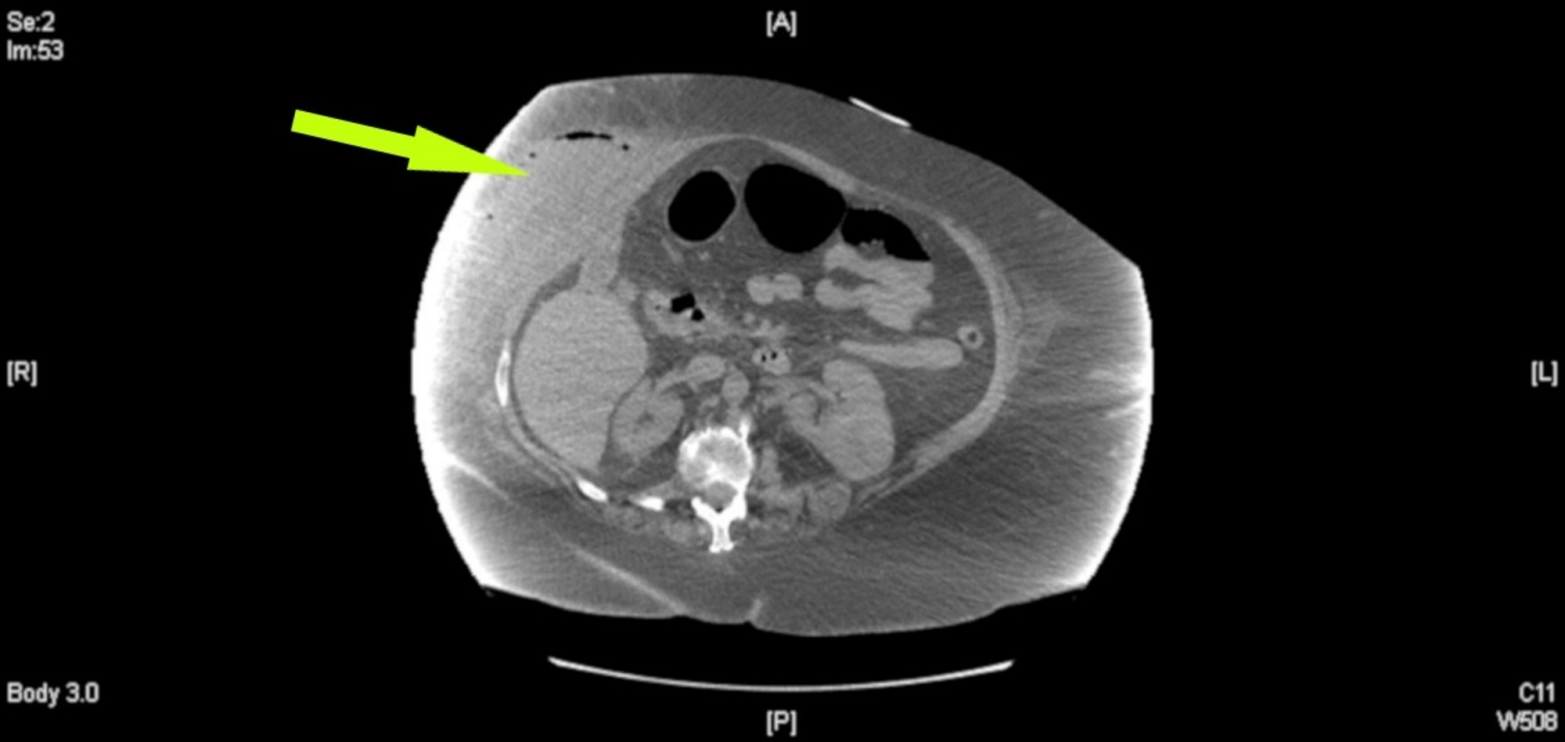
Additional axial view of noncontrast computed tomography of abdomen. Arrow shows large subcutaneous fluid collection noted over right upper quadrant with associated fat stranding and air-fluid levels.

A 60-year-old morbidly obese, white female was admitted to the floor with one week of worsening shortness of breath, right upper quadrant (RUQ) pain, nausea, emesis, and a fever. General surgery was consulted after a large, fluctuant and erythematous mass was visualized on the RUQ abdominal wall. The patient noted a history of severe cholecystitis the year prior that was managed by a percutaneous cholecystostomy drain. After the drain was removed, she was lost to follow up. Initial laboratory evaluation revealed: leukocytosis with 91% neutrophils (white blood cell count 14,800, reference range 3500 - 10300 mm3), international normalized ratio of 2.51 (reference range 0.90-1.10), alkaline phosphatase of 162 (reference range 20-130 U/L); lactic acid, aspartate aminotransferase, and alanine aminotransferase were within normal limits. A computed tomography (CT) scan of the abdomen revealed a 14 cm x 5 cm abdominal wall fluid and air collection suspicious for an abscess in the RUQ as seen in Figures 1-2.

**Figure 3:**
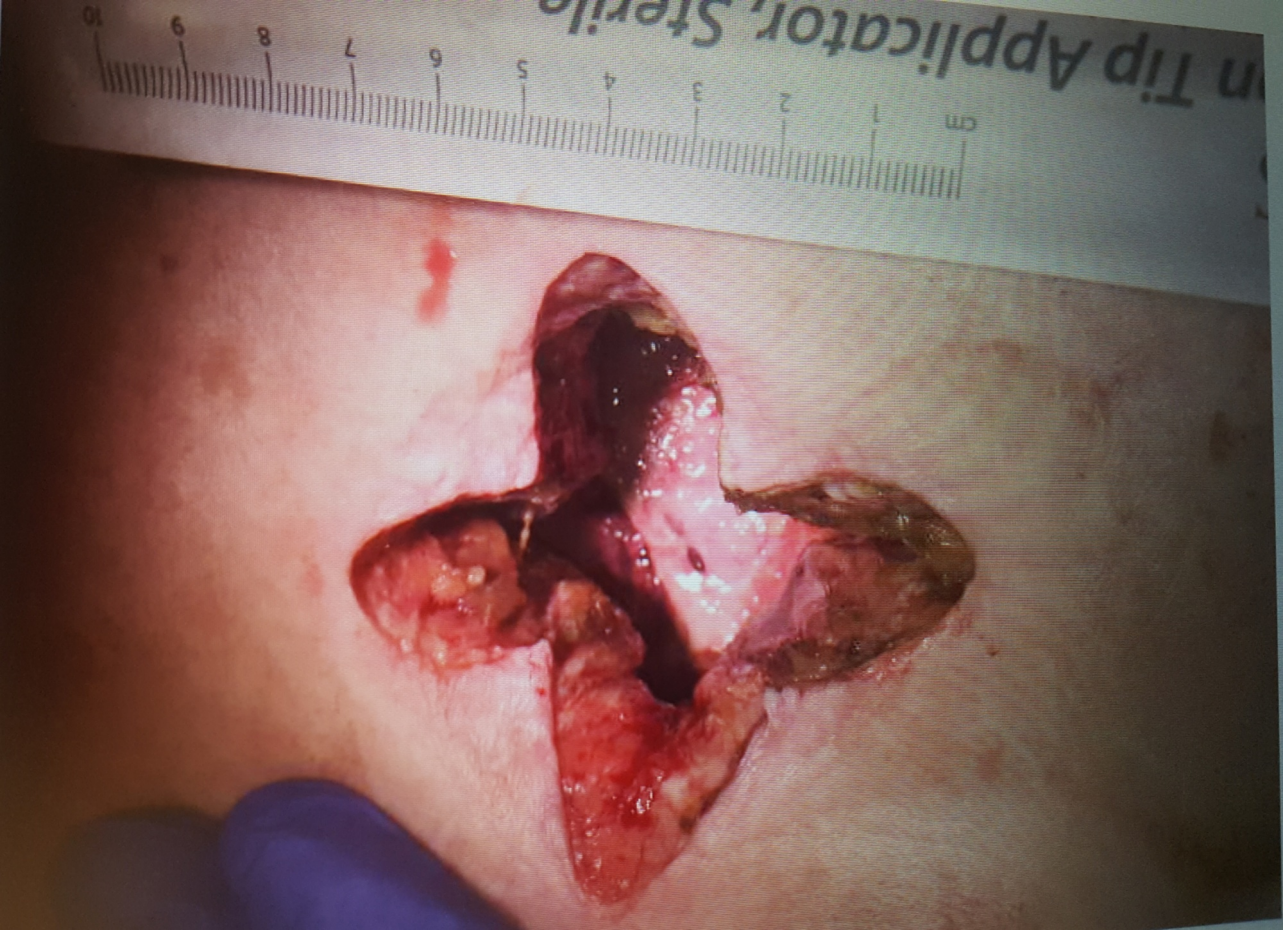
Right upper quadrant cruciate incision after pulse lavage with normal saline.

**Figure 4:**
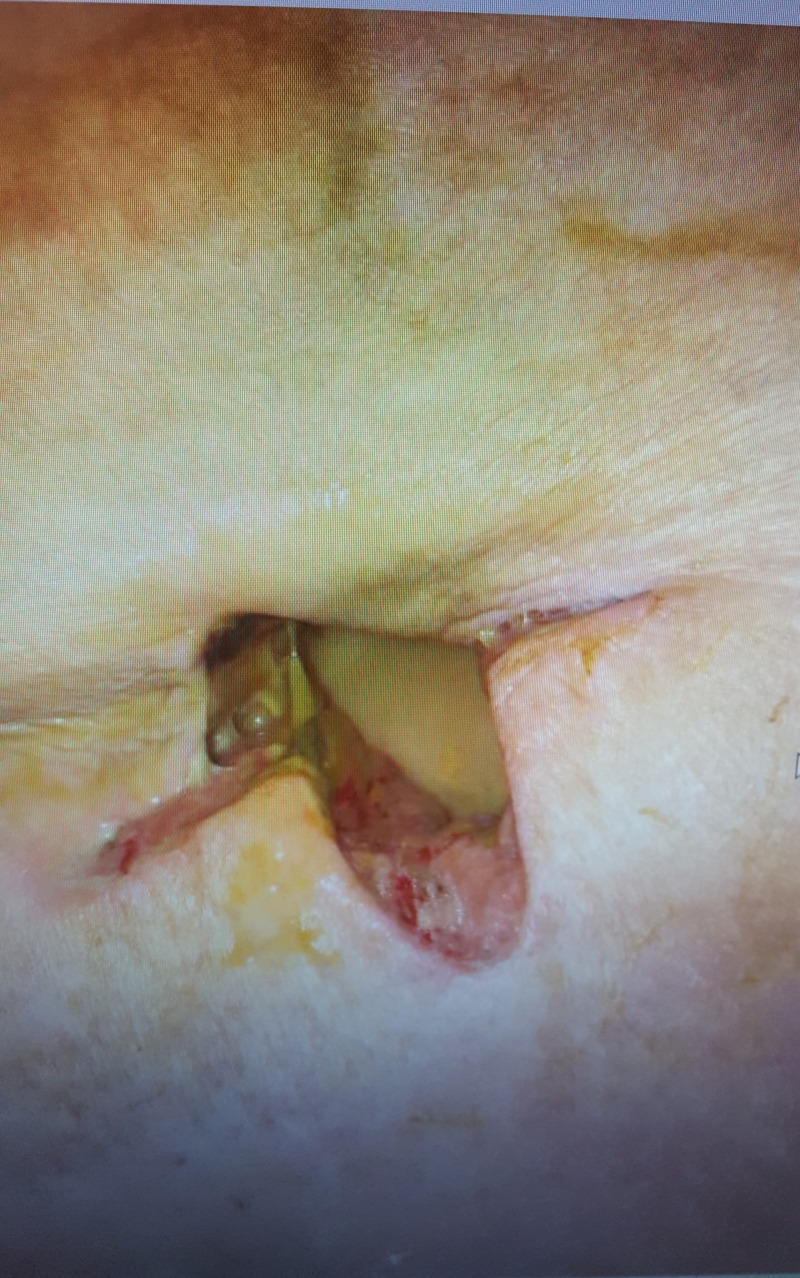
Incision site on postoperative day 7. Note the bilious appearing fluid draining from site during packing changes.

Incision and drainage (I&D) of the abscess were performed with a cruciate incision over the indurated RUQ of the abdomen as seen in Figure 3. One hundred and fifty milliliters of the purulent material was irrigated via pulsed lavage with normal saline. Intraoperative wound cultures revealed Escherichia coli along with Bacteroides fragilis, which were treated with ertapenem. The patient continued to improve with daily packing changes until day seven post operation, when she was noted to have yellow-green discharge draining from the wound site and increasing tenderness with packing changes. Figure 4 demonstrates the wound and discharge appearance. There was a concern that the fluid was bile rather than an infection due to the location and size of the initial abscess. A hepatobiliary iminodiacetic acid scan was performed but failed to demonstrate a biliary fistula tract. A second CT scan with oral contrast was ordered and after further review, it was noted that her gallbladder was severely contracted and located near the abscess site, which supported the idea of a CCF. This study was compared with a prior CT taken after percutaneous cholecystostomy tube placement in 2016. It was noted that the current abscess site was located near the drain placement site. Due to worsening drainage from the wound and the probable fistula formation, a robotic-assisted cholecystectomy with intraoperative cholangiogram was performed.

**Figure 5:**
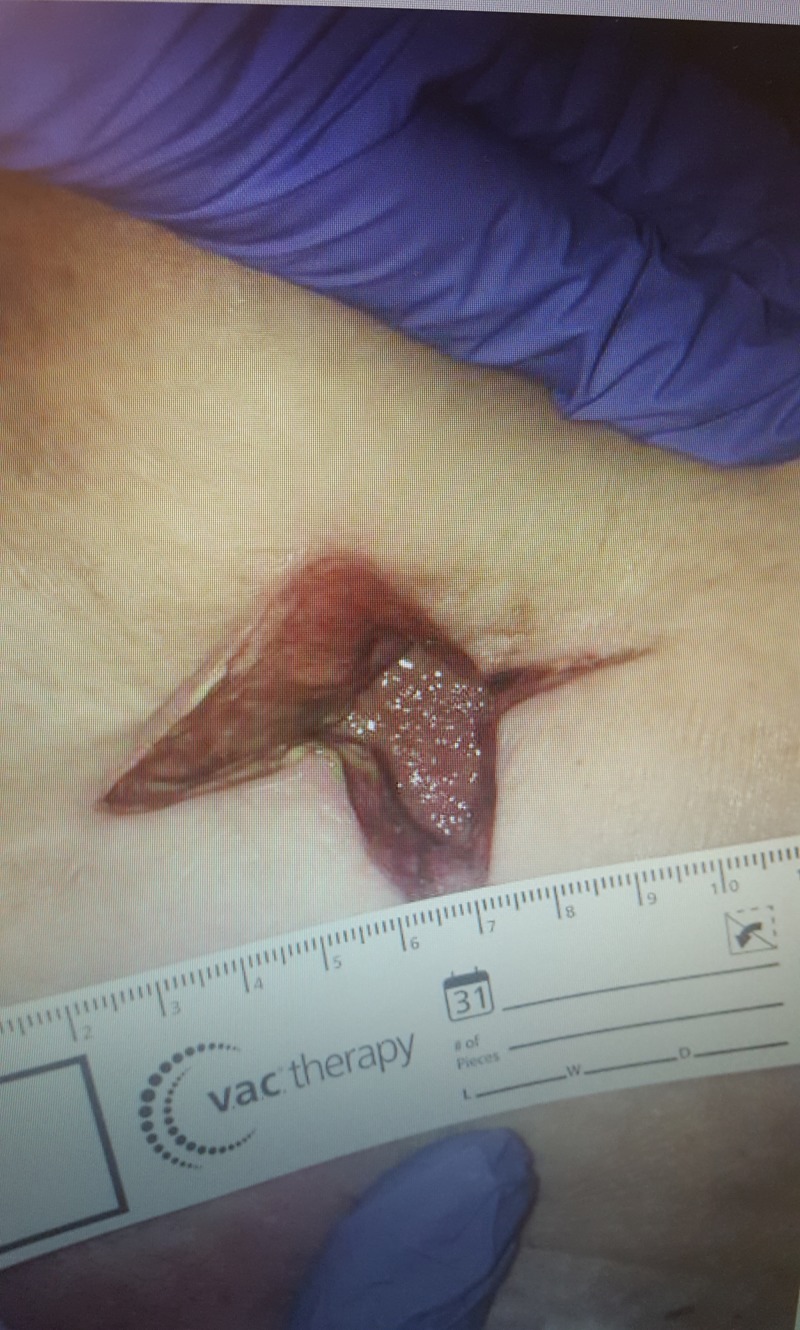
Incision site appearance day 7 after cholecystectomy with fistulotomy. Note early granulation tissue as well as no noted purulence or drainage.

Once inside the abdomen, dense adhesions were visualized in the RUQ of the peritoneal cavity along with a laterally adhered, contracted gallbladder. Two hours of adhesiolysis was performed due to the numerous dense adhesions in the RUQ. An intraoperative cholangiogram confirms the suspected anatomy and demonstrated mild distension of the common bile duct. The gallbladder was removed and sent for histopathological examination. It was 6.7 cm x 3.0 cm x 2.2 cm in size with associated cholelithiasis and chronic inflammatory changes. Postoperatively the patient remained stable throughout her course and received daily packing changes, ertapenem, and eventual negative pressure wound therapy placement. The patient was discharged on day 5 post-operation with subsided drainage, early granulation tissue, and improving wound erythema as seen in Figure 5. The patient was sent home with negative pressure wound therapy, education material, and an emphasis on the physician follow-up.

## Discussion

Biliary fistulas can be divided into the following categories: primary or secondary. Primary fistulas are generally associated with chronic cholelithiasis or biliary cancer, whereas secondary fistulas are associated with abdominal procedures. Biliary fistulas can further be broken down into external or internal categories, with the majority occurring internally. Internal connections include the duodenum, colon, stomach, jejunum, and common bile duct [2-3,6,10,12-15]. The CCF seen in this case, is a type of an EBF, which appears secondary to percutaneous drain placement with associated chronic cholecystitis.

Upon literature review, only one other case of a CCF associated with percutaneous cholecystostomy drain placement was noted [12]. In that study, the patient’s fistula was discovered due to visible purulent drainage and multiple gallstones protruding out of the previous percutaneous drain site. Subsequently, the patient had their drain removed during a separate hospitalization prior to elective cholecystectomy, allowing for continued inflammation and fistula formation, similar to our case. The patient in that study was successfully treated with a laparoscopic cholecystectomy as well. One major point of contrast between the two cases is how the fistula presented itself externally. Our case presented as an abdominal wall abscess rather than a draining sinus with extraversion of stones. Although there are other reports of CCFs presenting in as abdominal wall abscesses in the literature, this is the first one noted after percutaneous cholecystostomy tube placement and removal [4,7-8,15].

Percutaneous cholecystostomy drains are used in patients whose condition prevent them from having a cholecystectomy. They have been shown to be a safe and effective temporary treatment for patients with acute cholecystitis with severe comorbid conditions. Generally, the placement of these drains reduce inflammation and manage comorbidities prior to definitive treatment by cholecystectomy [16-17]. Most patients are scheduled for and follow up with their surgeons for a cholecystectomy during which they remove the drain. This unfortunately wasn’t seen in this patient as she had the perfect nidus for a fistula, a preformed tract with a continually inflamed gallbladder. Known complications of percutaneous cholecystostomy placement include drain dislodgement, biliary peritonitis, pneumothorax, and colonic perforation [16-17].

CCFs are generally treated with cholecystectomy, but some are left to close on their own depending on the patient’s comorbidities [18-19] In this case, due to the chronic nature of her gallbladder inflammation and recurrence of drainage from her abscess, she was treated via robotic-assisted cholecystectomy. This minimally invasive approach to cholecystectomy with CCF removal is associated with fewer risks to the patient compared with an open procedure and was preferred in this case due to the patient’s extensive morbid obesity and comorbidities.

## Conclusions

Although CCFs were historically a common outcome of gallbladder disease, they are now rarely seen due to improvements in the identification and treatment of gallbladder pathology. In this report, we presented a 60-year-old morbidly obese female who developed a CCFs secondary to continued cholecystitis with an associated preformed tract from prior cholecystostomy drain placement. The authors of this study hope that this case presentation will educate other clinicians to explore the diagnosis of an EBF when presented with a RUQ abdominal abscess.
